# Simultaneous involvement of 11q23 translocation resulting in chimeric *MLL-AFF1* and a second translocation [t (9;21) (p13; p11.2)] in an infant acute lymphoblastic leukemia patient at relapse: A case report

**DOI:** 10.1097/MD.0000000000010874

**Published:** 2018-05-25

**Authors:** Guangming Liu, Xianglan Lu, Young Mi Kim, Xianfu Wang, Shibo Li, Yuanyuan Liu

**Affiliations:** a Department of Gastroenterology, The First Hospital of Jilin University, Changchun, People's Republic of China; b Department of Pediatrics, University of Oklahoma Health Sciences Center, Oklahoma City, OK.

**Keywords:** FISH, infant ALL, *MLL*-*AFF1*, t (4 ;11 ;11), t (9 ;21)

## Abstract

**Rationale:**

Three-way translocations occasionally occur in *MLL-AFF1* fusion and other fusion gene. However, the complex chromosomal rearrangements in the study were the first report.

**Patient concerns:**

We present novel cryptic and complex chromosomal rearrangements [der (21) t (9; 21) (p13; p11.2)] in an infant patient with relapsed acute lymphoblastic leukemia (ALL).

**Diagnoses:**

The diagnosis was based on morphologic, cytochemical, and immunophenotypic criteria proposed by the French-American-British Committee, and karyotype, fluorescence *in situ* hybridization, array comparative genomic hybridization.

**Interventions:**

The patient was given chemotherapy with standard protocol for ALL.

**Outcomes:**

The patient had unfavorable prognostic outcome based on the cytogenetic and molecular cytogenetic markers. After short remission, the patient relapsed.

**Lessons:**

*MLL-AFF1*, resulting from t(4;11)(q21;q23), is regarded as the hallmark of infant t(4;11) pre-B/mixed B-ALL. It is associated with a dismal prognosis and the multiple-way translocation involving chromosomes 4, 11 and 11 may function as an enhancer.

## 1 Introduction

In acute lymphoblastic leukemia (ALL) patients, the human mixed lineage leukemia gene (*MLL*) often undergoes chromosomal rearrangements at the chromosome band 11q23, which leads to unique clinical and biological features and unfavorable prognosis.^[1,2]^ Overall, *MLL* rearrangements are found in ∼10% of human leukemia cases. However, up to 80% of infant ALL patients younger than 1 year and most patients with ALL linked to treatment with DNA topoisomerase II inhibitors carry such rearrangements.^[3]^ So far, *MLL* has been found to be involved in >100 different translocations in ALL, and >60 translocation partner genes have been molecularly characterized.^[4]^*AFF1 (*also known as *AF4)* is the most common partner gene, and *MLL-AFF1* fusion occurs in 46% of infant acute *ALL* patients. ^[4]^ The *AFF1* gene is fused with the *MLL* gene mostly by a balanced reciprocal translocation and occasionally by more complex chromosomal rearrangements such as 3-way translocations.^[5,6]^ Complex translocation, which also occurs in the majority of other classical translocations, such as t (11;19), t (12;21), and t (15;17), usually produces the same fusion gene that is typical of the simple balanced translocation.^[7–9]^

Identifying genetic alterations in infant ALL is critical for clinical diagnosis, classification, treatment, and prognosis. In this report, we present novel cryptic and complex chromosomal rearrangements [der (21) t (9; 21) (p13; p11.2)] in an infant patient with relapsed ALL.

This study was approved by the institutional review board (IRB) of the University of Oklahoma Health Sciences Center (IRB number: 6299; Oklahoma City, OK). Informed consent was obtained from the patient for publication of this case report and accompanying images.

## 2 Case report

A 2-month-old male, suffering fever, diarrhea, and vomiting, was admitted to the Health Sciences Center of the University of Oklahoma in 2014. Blood tests were performed immediately, and the results were as follows: hemoglobin, 9.6 g/L; leukocyte count, 33.1 × 10^9^ cells/L (neutrophils 6%, lymphocytes 33%, monocytes 7%, and blasts 50%); and platelets, 186 × 10^9^ cells/L. Bone marrow aspiration was performed and showed that the bone marrow was hypercellular with 50% blast cells. Also, the leukemic cells were negative for both myeloperoxidase and Sudan black B. Flow cytometric immunophenotypic analysis showed the leukemic cells were CD19(+), CD34(+), CD38(+), HLDR (+), moderately CD45(+), and partially CD15(+). There was no co-expression of CD10, CD20, surface immunoglobulin, CD13, CD33, CD117, or T-cell markers. No hepatomegaly or splenomegaly was observed. The patient was diagnosed with ALL, pre-B phenotype, based on the laboratory findings described above. After relapse, flow cytometric analysis was repeated and it showed similar marker patterns, CD19(+), CD34(+), CD38(+), HLDR (+), moderately CD45(+), and partially CD15(+). There was no co-expression of CD10, CD20, surface immunoglobulin, CD13, CD33, CD117, or T-cell markers.

**Figure 1:**
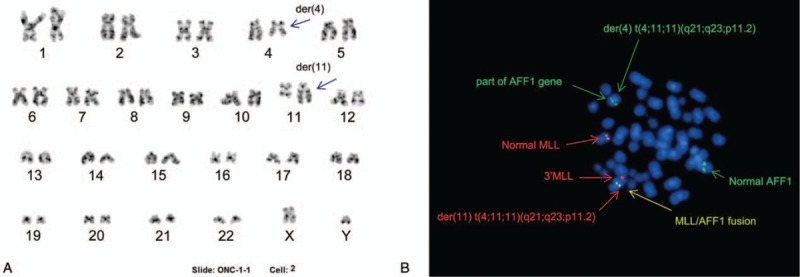
(A) Karyotype with 46, XY, t (4;11;11) (q21; q23; p11.2) at initial diagnosis; (B) Metaphase fluorescence in situ hybridization using DNA-probe *AFF1/MLL* (Abbott) dual-color dual-fusion probe indicated the 3’*MLL* gene was on the short arm of der (11) and a *MLL-AFF1* fusion signal was observed on the long arm of the same der(11), with part of the *AFF1* gene on the long arm of der(4).

Chromosome analysis of the bone marrow sample showed a 3-way translocation t (4;11;11) (q21;q23;p11.2), which resulted from translocation of the chromosome 4q21 segment to 11q23 and juxtaposition of the 11p11.2 segment to 4q21 (Fig. 1A). One of the 2 chromosome 11 had 2 breakpoints, with 1 on each arm. Breakpoints at 4q21 and 11q23 prompted us to search for a *AFF1* and *MLL* rearrangement; however, fluorescence in situ hybridization (FISH) with a *MLL/AFF1* dual-color dual-fusion probe revealed that only one *MLL-AFF1* fusion signal was on the derivative chromosome 11, the distal part of the *MLL* gene was translocated to the short arm of the same derivative chromosome 11 on 11p11.2, and the proximal part of the *AFF1* gene remained on the long arm of the derivative chromosome 4 (Fig. 1B). The above findings indicated that the patient had a variant translocation t (4;11) (q21;q23), having a new partner breakpoint on 11p11.2.

After receiving induction chemotherapy for 4 weeks, the patient achieved complete cytogenetic remission and the rearrangement of *AFF1* and *MLL* was no longer detectable by FISH. After 10 months, the blood tests were repeated and revealed: hemoglobin, 11.5 g/L; leukocyte count, 2.37 × 10^9^ cells/L; and platelets, 220 × 10^9^ cells/L. A peripheral smear study was repeated because of delay in count recovery and showed 41% blasts, and because of a high number of peripheral blasts, the patient was suspected of relapse. Bone marrow aspiration revealed 80% blasts and a paucity of normal cells. The results of both the peripheral smear and bone marrow study were consistent with marrow relapse of leukemia.

**Figure 2:**
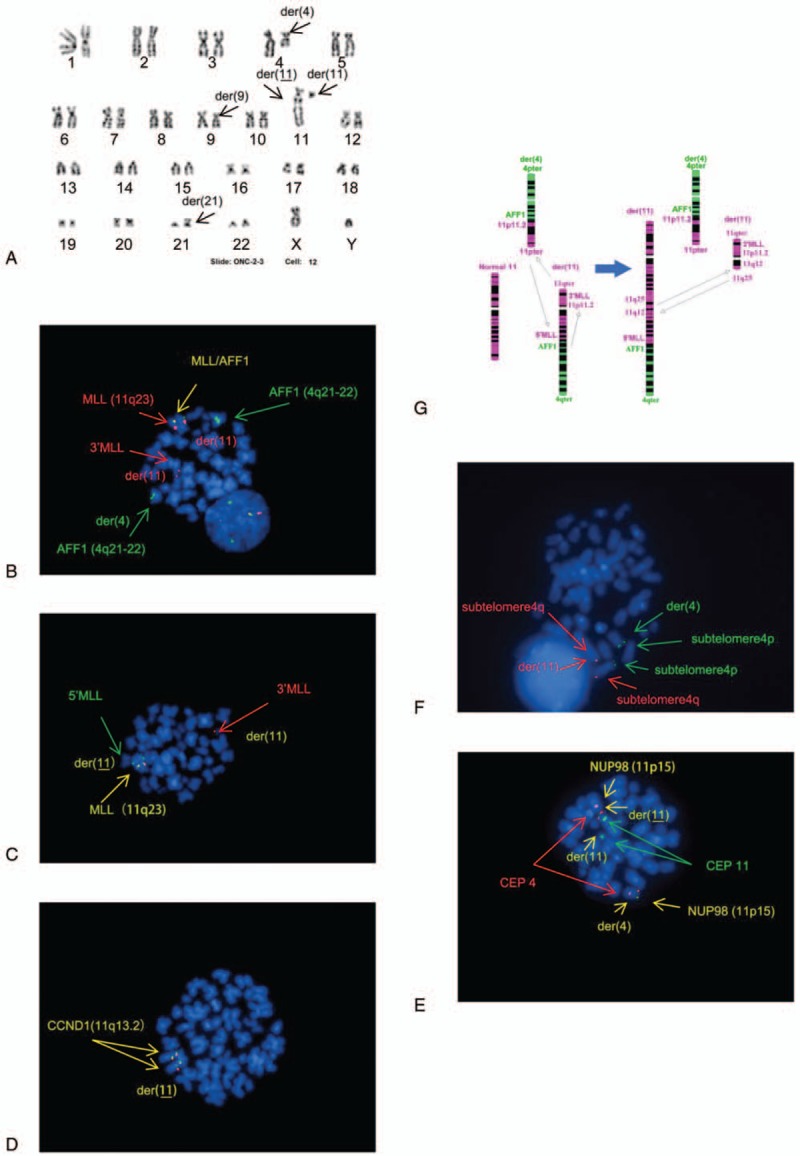
(A) Karyotype at relapse. (B) Metaphase fluorescence in situ hybridization (FISH) using DNA-probe *AFF1/MLL* (Abbott) dual-color dual-fusion probe indicated one normal *MLL* signal and *MLL-AFF1* fusion signal were on the homologous der (11), the 3’*MLL* signal was on the der (11), and part of the *AFF1* gene was on the long arm of der (4). (C) Metaphase FISH using DNA-probe *MLL* (Abbott) break apart probe indicated *3’MLL* was on the der(11), and *5’MLL* as well as the normal *MLL* gene were on the homologous der(11). (D) Metaphase FISH using DNA-probe *CCND1* (Abbott) break apart probe indicated both *CCND1* genes were on the homologous der(11). (E) Metaphase co-hybridization with FISH DNA-probe *NUP98* (Abbott) break apart probe labeled with Spectrum Orange and *CEP4* (Abbott) labeled with Spectrum Red and *CEP11* (Abbott) labeled with Spectrum Green indicated that one *NUP98* signal was on der (4), and the other *NUP98* signal was on the homologous der (11). (F) Metaphase co-hybridization with FISH DNA-probe subtelomere4q (Abbott) labeled with Spectrum Red and subtelomere4p (Abbott) labeled with Spectrum Green indicated that one subtelomere 4p signal was on der (4), and one subtelomere 4q signal was on the homologous der (11). (G) Schematic illustration of the chromosomal changes between chromosomes 4 and 11 from initial diagnosis to relapse.

We therefore characterized the cryptic and complex chromosomal rearrangements in this patient at relapse by both conventional chromosomal analysis and FISH assays. Most cells analyzed (14/20) by conventional chromosome analysis had consistent, complex structural rearrangements. Although at initial diagnosis the patient had a 3-way translocation between chromosomes 4 and 11 that resulted in a derivative chromosome 11 with 2 breakpoints on both the long and short arms with a large segment of chromosome 4 attached to the long arm of chromosome 11, the results at relapse showed that the derivative chromosome 11 had broken at the 11q12 region and joined to the homologous derivative chromosome 11 at 11q25, resulting in 2 derivative chromosomes 11. In other words, the leukemic cells did not have a single normal chromosome 11 (Fig. 2A). FISH assays showed that the distal part of the *AFF1* gene was moved to the long arm of chromosome 11 (11q23), where the *MLL* gene is located, and then with a breakpoint at 11q12, translocated to the end of the long arm of the homologous derivative chromosome 11 at 11q25. The rest of the *AFF1* gene remained on the long arm of the derivative chromosome 4; thus, the *MLL-AFF1* fusion signal and the normal *MLL* gene were both on the homologous derivative chromosome 11, and another part of the *MLL* gene was on the derivative chromosome 11 (Fig. 2B). Next, hybridization with a *MLL* break apart probe revealed that the normal *MLL* gene and the *5’MLL* gene were on the homologous derivative chromosome 11, and the *3’MLL* gene was on the derivative chromosome 11 (Fig. 2C). Hybridization with a *CCND1(11q13.2)* break apart probe revealed that both *CCND1* genes were on the homologous derivative chromosome 11 (Fig. 2D), indicating that the breakpoint on the derivative chromosome 11 was between 11q13.2 and the centromere. Furthermore, co-hybridization with FISH DNA probes *NUP98(11p15)*/*CEP4*/*CEP11* indicated that one *NUP98* gene was on the long arm of the derivative chromosome 4, whereas the other *NUP98* gene was on the short arm of the homologous derivative chromosome 11 (Fig. 2E). Co-hybridization with FISH DNA-probe subtelomere *4q* and *4p* indicated that 1 subtelomere *4p* signal was on the derivative chromosome 4, and 1 subtelomere *4q* signal was on the homologous derivative chromosome 11 (Fig. 2F). The above assays revealed multiple translocations in different directions, as summarized in Figure 2G. Five breakpoints on three chromosomes were involved in these complex, multidirectional translocations. Starting from chromosome 4q21, the rearrangement followed this sequence: 4q21→11q23→11p11.2 →4q21 and then 11q12⇄11qter.

**Figure 3:**
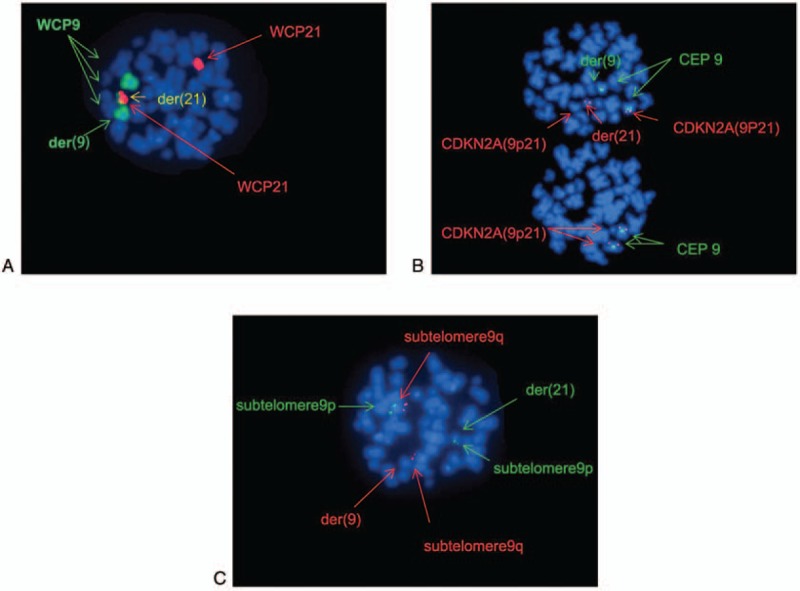
(A) Co-hybridization of whole chromosome 9 painting probe (Abbott) labeled with Spectrum Green and whole chromosome 21 painting probe (Abbott) labeled with Spectrum Red revealed that the short arm of chromosome 9 translocated to chromosome 21, forming t (9;21) (p13; p11.2). (B) Metaphase fluorescence in situ hybridization (FISH) using DNA-probe *LSI CDKN2A(9p21)* (Abbott) labeled with Spectrum Red and *CEP9* (Abbott) labeled with Spectrum Green indicated a *CDKN2A* gene was on der (21). (C) Metaphase FISH using DNA-probe subtelomere *9q* (Abbott) labeled with Spectrum Red and subtelomere *9p* (Abbott) labeled with Spectrum Green indicated a subtelomere *9p* signal was on der (21).

**Figure 4:**
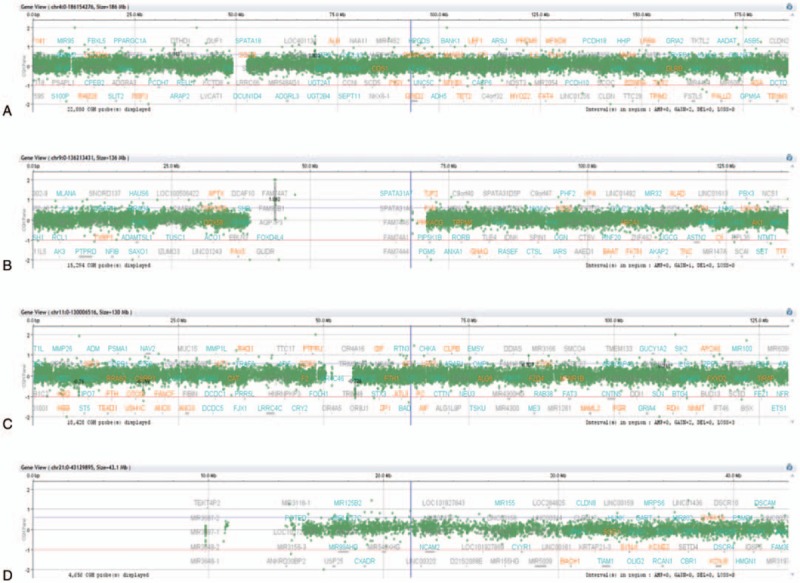
Results of the oligoarray array comparative genomic hybridization using NimbleGen SegMNT and the RefSeq genes in the abnormal region (University of California, Santa Cruz genome browser hg19). The *y* axis indicates a gain or loss of genetic material, whereas the *x* axis indicates the genomic position of each feature on the chromosome. No gain or loss was observed on chromosome 4 (A), chromosome 9 (B), chromosome 11 (C), or chromosome 21 (D).

We also identified a second translocation between chromosomes 9 and 21 [der (21) t(9;21) (p13;p11.2)] in the karyotype at relapse (Fig. 2A). FISH analysis was then performed to further characterize these structural changes, using multiple DNA probes. Co-hybridization of whole chromosome 9 and 21 painting probes revealed that a part of the short arm of chromosome 9 was moved to chromosome 21, forming a derivative chromosome 21. Next, hybridization with *CDKN2A(9p21)*/*CEP9* probe revealed a *CDKN2A* gene on derivative chromosome 21, and co-hybridization with subtelomere *9p* and *9q* indicated that subtelomere *9p* was on the short arm of derivative 21 (Fig. 3A–C). The resulting karyotype was: 46, XY,der(4)(4pter→4q21::11p11.2→11pter),t(9;21)(p13;p11.2),der(11) (11qter → 11q23:: 11p11.2→11q12::11q25),der(11)(11pter→11q25:: 11q12→11q23:: 4q21→4qter) (Fig. 2A, 2G). Further array CGH analyses on a relapse specimen revealed no gain or loss on chromosomes 4, 9, 11, and 21 (Fig. 4).

## 3 Discussion

We have described a unique pediatric ALL case, in which the leukemic cells harbored a novel 3-way translocation t(4;11;11) (q21;q23;p11.2) at initial diagnosis and multiple translocations in different directions involving chromosomes 4q21, 11p11.2, 11q12, 11q23, and 11q25 in addition to t(9;21)(p13;p11.2) at relapse. This complex translocation resulted in the fusion of *AFF1* to *MLL* on the der (11), just as with a classical t(4;11) (q21;q23).

The 11q23 region, the genetic hallmark of most infant B-ALL, is frequently rearranged in de novo and therapy-related AML and ALL, mostly in reciprocal exchanges with various translocation partners.^[7]^ To date, 104 translocation fusion sites have been identified, and 64 of them have been defined at the molecular level.^[4,7]^ Besides several simple translocations affecting 11q23, many complex rearrangements, involving multiple chromosomes, have been characterized.^[4,10]^ Additionally, Meyer et al^[4]^ showed reciprocal *MLL* gene fusions, which presented fusions of the 3’ portion of the *MLL* gene and a third partner gene because of complex rearrangements. Most these *MLL* fusions are unable to generate fusion proteins because of recombination between noncompatible introns or head-to-head fusions.^[4]^*AFF1*, *MLLT3*, *MLLT1*, *MLLT11*, and *ELL*, which are most frequent involvement with 3-way translocations, are considered the most common partners identified in leukemia with *MLL* rearrangement.^[11]^ Also, it has been reported that 17% of cases display complex rearrangements between chromosomes 4, 11, and a third chromosome in all *MLL-AFF1* fusion-positive cases.^[5]^

*MLL-AFF1,* resulting from t(4;11)(q21;q23), is regarded as the characteristic of infant t(4;11) pre-B/mixed B-ALL and associated with short latency, central nervous system infiltration, therapy of refractory ALL, and a dismal prognosis.^[12–14]^ Whole-genome sequencing studies reported a silent mutational landscape in MLL rearrangement infant B-ALL, suggesting that a single driver mutation (MLL-rearrangement) suffices to spawn this aggressive B-ALL.^[15,16]^ However, whether MLL-AFF1 fusion alone plays a role as a single “big-hit” sufficient to cause B-ALL is still unclear.^[17]^ It is reported that the MLL-AFF1 malignant potential could not be investigated because der(11)-encoded MLL-AFF1 fusion failed to generate an immortalized colony in semi-solid agar or engraftment in recipient mice.^[5,18]^

The translocation involving chromosomes 4 and 11 generates a fusion gene of *AFF1* from chromosome 4 and *MLL* from chromosome 11 and is considered as the initial event in the present case. The rearrangement involving the short arm of chromosome 9 occurred subsequently, leading to translocation between chromosome 9 and chromosome 21, the significance of which is unclear in the present case. One possibility is that t (9;21) is commonly present in patients with ALL, but has not been recognized before. To our knowledge, the t (9;21) has never been described in infant ALL before. Its impact on prognosis and relapses remains to be investigated. We speculate it is highly possible that this series of chromosomal changes may act cooperatively in leukemogenesis. Also, ^[19,20]^ multiple cases with variant, recurrent translocations, such as t(1;9;22), have documented with unfavorable prognosis, which the third chromosome involvement may play the role.

In summary, we report a rare case of an infant ALL patient with a novel three-way translocation t (4;11;11) (q21; q23; p11.2) at initial diagnosis and multiple translocations between chromosomes 4 and 11 in addition to t (9;21) (p13; p11.2) at relapse. We speculate these sequential events triggered the initiation and progression of infant ALL. These results may explain the patient's rapid clinical course and contribute to defining prognostic factors and developing treatment guidelines for ALL.

## Author contributions

**Conceptualization:** Yuanyuan Liu.

**Data curation:** Yuanyuan Liu.

**Formal analysis:** Shibo Li, Xianfu Wang.

**Funding acquisition:** Shibo Li.

**Investigation:** Shibo Li, Young Mi Kim.

**Methodology:** Shibo Li, Young Mi Kim, Xianfu Wang.

**Project administration:** Shibo Li.

**Resources:** Shibo Li, Xianglan Lu.

**Software:** Shibo Li, Xianglan Lu, Young Mi Kim, Xianfu Wang.

**Supervision:** Xianglan Lu.

**Validation:** Xianglan Lu.

**Visualization:** Xianglan Lu.

**Writing – original draft:** Guangming Liu.

**Writing – review & editing:** Guangming Liu.
